# Development and bin mapping of gene-associated interspecific SNPs for cotton (*Gossypium hirsutum* L.) introgression breeding efforts

**DOI:** 10.1186/1471-2164-15-945

**Published:** 2014-10-30

**Authors:** Amanda M Hulse-Kemp, Hamid Ashrafi, Xiuting Zheng, Fei Wang, Kevin A Hoegenauer, Andrea BV Maeda, S Samuel Yang, Kevin Stoffel, Marta Matvienko, Kimberly Clemons, Joshua A Udall, Allen Van Deynze, Don C Jones, David M Stelly

**Affiliations:** Department of Soil and Crop Sciences, Texas A&M University, College Station, Texas USA; Genetics Graduate Program, Texas A&M University, College Station, Texas USA; Seed Biotechnology Center, University of California, Davis, California USA; Monsanto Company, Molecular Breeding Technology, 700 Chesterfield Parkway West, CC224-B, Chesterfield, MO 63017 USA; CLC Bio, a QIAGEN Company, Davis, CA USA; Plant and Wildlife Science Department, Brigham Young University, 295 WIDB Provo, UT USA; Cotton Incorporated, Cary, NC USA

**Keywords:** Cotton, *Gossypium barbadense*, *Gossypium tomentosum*, *Gossypium mustelinum*, *Gossypium armourianum*, *Gossypium longicalyx*, RNA-seq, Interspecific SNP

## Abstract

**Background:**

Cotton (*Gossypium spp.*) is the largest producer of natural fibers for textile and is an important crop worldwide. Crop production is comprised primarily of *G. hirsutum* L., an allotetraploid. However, elite cultivars express very small amounts of variation due to the species monophyletic origin, domestication and further bottlenecks due to selection. Conversely, wild cotton species harbor extensive genetic diversity of prospective utility to improve many beneficial agronomic traits, fiber characteristics, and resistance to disease and drought. Introgression of traits from wild species can provide a natural way to incorporate advantageous traits through breeding to generate higher-producing cotton cultivars and more sustainable production systems. Interspecific introgression efforts by conventional methods are very time-consuming and costly, but can be expedited using marker-assisted selection.

**Results:**

Using transcriptome sequencing we have developed the first gene-associated single nucleotide polymorphism (SNP) markers for wild cotton species *G. tomentosum, G. mustelinum, G. armourianum* and *G. longicalyx*. Markers were also developed for a secondary cultivated species *G. barbadense* cv. 3–79. A total of 62,832 non-redundant SNP markers were developed from the five wild species which can be utilized for interspecific germplasm introgression into cultivated *G. hirsutum* and are directly associated with genes. Over 500 of the *G. barbadense* markers have been validated by whole-genome radiation hybrid mapping. Overall 1,060 SNPs from the five different species have been screened and shown to produce acceptable genotyping assays.

**Conclusions:**

This large set of 62,832 SNPs relative to cultivated *G. hirsutum* will allow for the first high-density mapping of genes from five wild species that affect traits of interest, including beneficial agronomic and fiber characteristics. Upon mapping, the markers can be utilized for marker-assisted introgression of new germplasm into cultivated cotton and in subsequent breeding of agronomically adapted types, including cultivar development.

**Electronic supplementary material:**

The online version of this article (doi:10.1186/1471-2164-15-945) contains supplementary material, which is available to authorized users.

## Background

Cotton (*Gossypium spp.*) is the leading natural fiber crop worldwide and an important contributor to the economies of nearly 100 countries. The genus *Gossypium* is also an important model species for polyploidy and the biological processes of cell wall elongation and cellulose biosynthesis in fiber cells. This clade consists of approximately 45 diploid species and five allotetraploid species. Genomes of the allotetraploid species have 52 chromosomes (2n = 4x = 52) and are believed to have originated from a single polyploidization event between an A-genome diploid (n = 2x = 26) and a D-genome diploid (n = 2x = 26) approximately 1–2 million years ago [1]. The five allotetraploid species share a basic AD genome architecture. Chromosomes of the *G. hirsutum* genome ([AD]_1_) have been numbered according to their evolutionary origins and meiotic pairing relationships. Chromosomes 1–13 comprise the “A” sub-genome (A_T_) that originated from the extinct A-genome diploid ancestor and chromosomes 14–26 comprise the “D” sub-genome (D_T_) that originated from the extinct D-genome diploid ancestor. There are four major cultivated species wordwide, two diploids *G. arboreum* (A_2_ genome) and *G. herbaceum* (A_1_) and two allotetraploids *G. hirsutum* L. or Upland cotton and *G. barbadense* L. ([AD]_2_), extra long-staple Pima, Egyptian cotton or Sea Island cotton. Upland cotton cultivation represents over 95% of the fiber produced worldwide due to its high yield, but generally Pima cotton the next most cultivated cotton, exhibits longer, stronger, and finer fiber.

Upland cotton has a very narrow genetic base due to multiple bottleneck events including, polyploidization, domestication and continuous selection. It has been suggested and experimentally tested that current Upland cultivars descend from only about a dozen introgressions and therefore exhibit an extremely small amount of diversity [2, 3]. With such small diversity in elite cotton germplasm, it is unlikely that sufficient variation for agronomically important traits, such as, fiber properties, yield, disease and insect resistance, drought tolerance and changing atmospheric conditions will be found within currently available elite breeding germplasm. Wild cotton species harbor large numbers of unique genes, which upon introgression may provide novel diversity for genetic improvement.

Diploid cotton species have been shown to have many disease and insect resistance traits, as well as improved fiber characteristics. The diploid *G. longicalyx* Hutch and Lee (F_1_) is the only member of the F-genome clade and is native to Africa. It has been shown to have resistance to pathogens, such as reniform nematode [4], and to have beneficial genes for fiber quality [5]. The diploid *G. armourianum* Kearney (D_2–1_) belongs to the D-genome clade and is a wild species found in Mexico. It has been shown to exhibit resistance to the whitefly [6], which is the vector for many cotton pathogens such as the leaf curl virus [7]. The diploid species exhibit a large range of relative genome sizes. Due to the difference in chromosome number between diploids and cultivated cotton, methods to move genes from diploids into cultivated tetraploid cotton using synthetic tri-species hybrids have been devised to introgress desired diploid segments through breeding [8, 9].

While crossing cultivated tetraploid cotton directly with diploid species is difficult, allotetraploid species can be easily interbred and then backcrossed to move desired segments into cultivated material. The tetraploid *G. tomentosum* Nuttal ex Seeman originates from the Hawaiian islands and produces a small amount of short, reddish brown fiber. *G. tomentosum* has been found to show resistance against the cotton leaf hopper, *Amrasca biguttula biguttula*, and thrips, *Frankliniella occidentalis*[6]. The tetraploid *G. mustelinum* Meers ex Watt is from Brazil and also produces a small amount of lint. Using HPLC analysis, *G. mustelinum* has been shown to have the highest leaf concentrations of terpenoid aldehydes that affect insect resistance [10]. *G. barbadense* originates from South America and is a cultivated species which represents about five percent of the annual worldwide fiber crop. This tetraploid exhibits excellent fiber quality characteristics for fiber length, micronaire and high strength relative to *G. hirsutum*.

Many of the mapping efforts in cotton have consisted of interspecific biparental populations of *G. hirsutum × G. barbadense* which offers a higher polymorphism rate than intraspecific crosses, and segregation for superior fiber quality characteristics*.* Moderate density linkage maps have been created using restriction fragment length polymorphisms (RFLPs) [11, 12], amplified fragment length polymorphisms (AFLPs) [13] and simple sequence repeats (SSRs) [14, 15]. SSRs have also been used for wide-cross whole-genome radiation hybrid (WWRH) mapping for production of syntenic groups [16, 17]. A consensus map was recently created which integrated all of the previous mapping efforts [18]. While single nucleotide polymorphisms (SNPs) represent the most prevalent category of polymorphisms available within the genome, few studies have developed and mapped SNPs in cotton [14, 19]. SNP development efforts to-date have produced relatively few numbers of SNPs using different genome reduction methods in cultivated species [19–23].

An aspect of polyploid genomes that creates difficulties during SNP development is that there are two indistinguishable types of SNPs in polyploid sequence data: homeologous sequence variants or “homeo-SNPs” and traditional SNPs or “allele-SNPs” [24]. A catalogue of homeo-SNPs, which are differences between the A-genome and D-genome diploid species, was recently identified in *Gossypium* diploid and tetraploid genomes [25, 26]. In cotton tetraploids, five million homeo-SNPs were found between the A_T_ and D_T_ subgenomes, which was facilitated by recent publication of the reference genome sequence for *G. raimondii* (D_5_) [27]. The D_5_ genome is regarded as the closest living diploid relative to the D-genome ancestor of current AD-allotetraploid species [28]. It has been hypothesized that the catalogued homeo-SNPs can possibly be used to filter putative SNPs when sequence reads are aligned within the framework of the base-pair coordinates of reference diploid genomes [27, 29]. While homeo-SNPs may allow for separation of homeologous sequences, they are not directly applicable to breeding. As allele-SNPs identify polymorphisms within a haplotype, experimental assays can be developed to genotype individuals and track favourable and unfavourable alleles. Upon germplasm introgression from wild species, whether diploid or tetraploid, orthologous sequence variants become allele-SNPs. High-density interspecific allele-SNPs distributed across both sets of *G. hirsutum* chromosomes will be useful for breeders to efficiently introgress quantitative trait loci (QTLs) and track alleles in marker-assisted selection (MAS) of beneficial traits from donor species.

Traditionally, interspecific introgression breeding efforts are extremely time-consuming and require large amounts of effort and funds. Interspecific genetic introgression into *G. hirsutum* has thus far been constrained by the paucity of high-throughput genome-wide markers that would facilitate tracking of introgressed segments. The relative scarcity of SNPs in cultivated allotetraploid cotton reflects the difficulty of developing SNPs for its complex genome, comprised of large repetitive regions and homeologous content due to recent polyploidization. Here, we report a method utilizing the genomic reduction method of transcriptome sequencing to derive interspecific gene-associated SNPs between the genetic standard *G. hirsutum* TM-1, and five other species, including the genetic standard *G. barbadense* doubled haploid line 3–79, two allotetraploids *G. tomentosum* and *G. mustelinum,* and two diploids *G. armourianum* and *G. longicalyx.* These SNPs will be extremely beneficial for high-density interspecific mapping and will help revolutionize introgression breeding efforts by facilitating MAS-based introgression, genetic dissection, and gene utilization in cultivated cotton.

## Results

### SNP development

Utilizing the *G. hirsutum* (line TM-1) transcriptome assembly produced by Ashrafi et al. (in preparation) consisting of 72,450 contigs covering over 70 M bp with N50 of 1,100 bp, transcriptome sequence reads (Table 1) were aligned and utilized to identity and filter a total of 10,888 SNPs *in silico* for *G. barbadense* line 3–79 relative to *G. hirsutum* line TM-1. With the same bioinformatic pipeline, SNPs were also developed for *G. tomentosum* (9,520), *G. mustelinum* (10,988)*, G. armourianum* (26,974)*,* and *G. longicalyx* (38,217). Reads which mapped to multiple locations were randomly assigned to a single location to achieve higher mapping coverage with limited number of reads. Filtering included removal of theoretical homeo-SNP positions based on an index created by mapping back Illumina TM-1 reads to the assembly. All of the markers identified within a given species were classified according to surrounding polymorphisms for the same species. Marker classifications were based only on species-specific polymorphism data and determined independently for each species. “Class I” was a SNP in which no additional polymorphism was found to exist in the same contig. “Class II” was a SNP in which (an) additional polymorphism(s) was found within the same contig, but the additional polymorphism was outside of 50 base pairs (bp) of the marker. “Class III” was a SNP in which additional polymorphisms were found in the same contig and within 50 bp of the marker. The SNPs for *G. barbadense*, *G. tomentosum*, *G. mustelinum*, *G. armourianum* and *G. longicalyx* were classified according to these criteria (Table 2).

**Table 1:** **Transcriptome sequence information**

Species	Sample	Raw reads (#)	Trimmed reads (#)	Mapped reads (#)	Depth	Reference coverage (%)
*G. barbadense*	3-79	101,276,621	101,276,621	43,519,596	48.45	84%
*G. tomentosum*	19909036.05	63,119,599	63,118,203	38,439,408	31.77	87%
*G. mustelinum*	200508123.02	65,940,564	65,940,111	40,767,777	33.07	86%
*G. armourianum*	D2-1-6	53,279,426	53,279,087	20,266,019	20.90	68%
*G. longicalyx*	200908137.04	52,050,537	52,050,305	23,393,277	24.50	65%

Raw and processed read information of Illumina GA-II (Solexa) sequence generated from RNA-Seq libraries for *G. barbadense*, *G. tomentosum*, *G. mustelinum*, *G. armourianum*, and *G. longicalyx*.

**Table 2:** **List of unfiltered SNPs determined for all species**

	Class I	Class II	Class III	Total
*G. barbadense*	3,257	6,385	1,246	10,888
*G. tomentosum*	1,520	6,526	1,474	9,520
*G. mustelinum*	1,678	7,584	1,726	10,988
*G. armourianum*	7,331	14,523	5,120	26,974
*G. longicalyx*	14,546	18,960	4,711	38,217

Number of SNPs derived *in silico* relative to *G. hirsutum* inbred line TM-1 for species *G. barbadense*, *G. tomentosum*, *G. mustelinum*, *G. armourianum*, and *G. longicalyx*. SNPs are classified into three categories, Class I are SNPs from contigs with no other SNP residing within the contig. Class II are SNPs from contigs that contain one or more additional SNP outside of the 50-bp flanking sequences (none within). Class III are SNPs from contigs that contain one or more additional SNPs within the 50-bp flanking sequences.

### Removal of redundant markers

**Figure 1:**
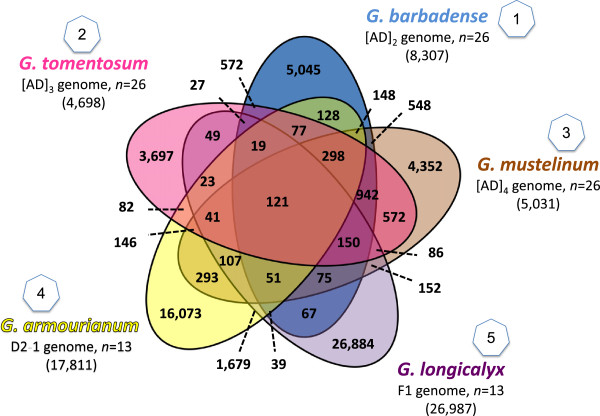
**Overlap of SNPs among species.** The overlap and specificity of the Class I and Class II SNPs for *G. barbadense* cv. 3–79, *G. tomentosum*, *G. mustelinum, G. armourianum,* and *G. longicalyx.*

SNPs that were redundant across species were reduced to a single instance by means of progressive comparisons (see Methods). The overlap with intraspecific *G. hirsutum* markers (Ashrafi et al. - in preparation) was low, e.g., only 3.3% or 367 markers of the *G. hirsutum*-*G. barbadense* SNPs were found to be redundant compared to the intraspecific SNPs. The overlap among the different species sets was plotted in a Venn Diagram, which revealed moderate levels of overlap among the three AD species (Figure 1). Following the stated progression, a total of 10,521 non-redundant *G. barbadense* SNPs were identified, 2,647 were Class I, 5,660 were Class II (Additional file 1), and 1,189 were Class III (Additional file 2). In addition, when the *G. barbadense* set was BLASTed against itself, 1,025 markers were redundant within the *G. barbadense* set (Additional file 3). SNPs of this redundant nature have been identified and listed separately for each species, so that they can be avoided (or targeted) for future species-specific studies on alternative splicing or gene family composition. For *G. tomentosum* 6,396 SNPs were retained, 811 in Class I, 3,885 in Class II (Additional file 4), and 1,107 in Class III (Additional file 2), while 593 redundant SNPs were listed separately (Additional file 5). A total of 6,663 SNPs were retained for *G. mustelinum*, 822 in Class I, 4,085 in Class II (Additional file 6), 1,107 in Class III (Additional file 2) and 592 redundant SNPs (Additional file 7). For *G. armourianum*, 5,723 Class I, 12,033 Class II (Additional file 8), 4,648 Class III (Additional file 2) and 2,425 redundant SNPs (Additional file 9) were obtained for a total of 24,829 SNPs. Lastly for *G. longicalyx* a total of 34,550 SNPs were identified, including 11,435 in Class I, 15,454 in Class II (Additional file 10), 4,309 in Class III (Additional file 2) and 3,352 SNPs which were redundant and listed separately (Additional file 11). In the final set of 62,555 non-redundant Class I and Class II SNPs for all of the five species, the transition to transversion ratio was 1.63 (38,763/23,792). Non-redundant SNPs were unique in the final set for the SNP and 50bp flanking sequences (now reference as SNP markers).

### Alignment of markers to D_5_-reference genome

**Figure 2:**
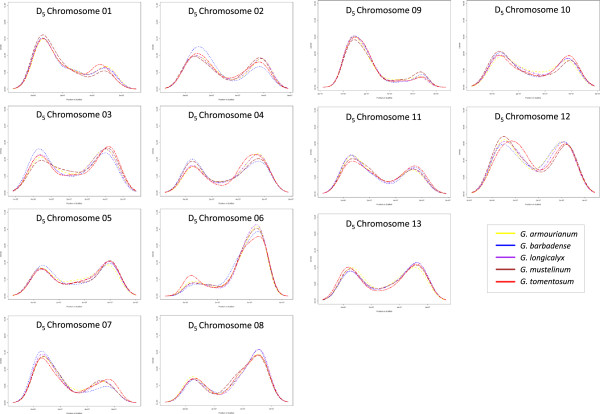
**Distributions of SNPs relative to** ***Gossypium raimondii*** **(D**_**5**_ **) draft genome.** All SNPs for each species were plotted according to BWA alignment positions (X-axis) across the *G. raimondii* (D_5_) draft genome over a sliding window in R. Density (Y-axis) is the proportion of the number of SNPs within a species-specific data set calculated over a sliding window. A figure was produced for each of the 13 scaffolds of the draft genome sequence. *G. barbadense* cv. 3–79 is shown in blue, *G. tomentosum* is shown in red, *G. mustelinum* is shown in brown, *G. armourianum* is shown in yellow, *G. longicalyx* is shown in purple.

A moderate share (75.87%) or 47,672 SNP markers in the final set could be aligned to the *Gossypium raimondii* (D_5_) diploid reference genome sequence. *G. armourianum* had the highest percentage of mapped markers, followed by *G. longicalyx,* and the three tetraploids *G. barbadense, G. tomentosum,* and *G. mustelinum*. Nearly all of the mapped markers (99.7%) aligned to one of the thirteen pseudo-chromosome scaffolds, and only 154 (0.3%) markers were aligned to unplaced scaffolds. A bimodal distribution across each D_5_-chromosome was observed when average density of markers was plotted (Figure 2).

### Validation of SNPs

Random sets of markers from the non-redundant final set of Class I and Class II SNPs were tested using KASP end-point assays (LGC Genomics, Beverly, MA, USA) from each species. As random sets of markers for each species were selected for screening prior to development of the final non-redundant set, some markers may have been tested on species other than the ones with which they were associated in the non-redundant list of SNPs (Additional files 2, 3, 4, 5, 6, 7, 8, 9, 10 and 11). This is due to the fact that some markers detected the same SNP between *G. hirsutum* and multiple species and was retained only once in the non-redundant list. The validation status of each marker for tested species is noted in columns B and C of Additional files 2, 3, 4, 5, 6, 7, 8, 9, 10 and 11.

**Figure 3:**
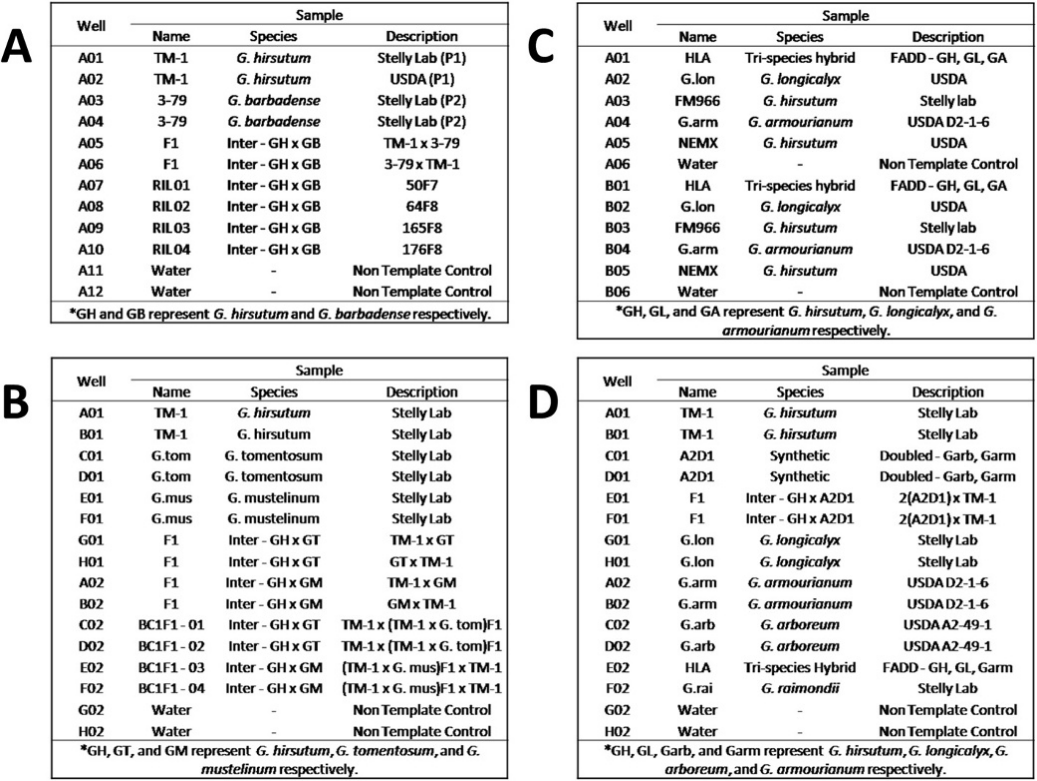
**KASP marker screening panels.** Screening panels containing control and mapping samples used for determining successful and unsuccessful markers via KASP assay genotyping. **(A.)** Panel used for screening markers derived from *G. barbadense*, “*G. barbadense* screening panel”. **(B.)** Panel used for screening markers derived from *G. tomentosum* and *G. mustelinum*. **(C.)** Panel used for screening markers derived from *G. longicalyx*. **(D.)** Panel used for screening markers derived from *G. armourianum*.

In the final non-redundant data sets, a total of 665 randomly selected markers were tested from the *G. barbadense* Class I and Class II set. Of these, 262 markers were tested on the “*G. barbadense* screening panel” (Figure 3) and 209 (79.8%) had clean clusters which allowed for scoring P1, P2 and F1 genotypes (successful markers). The remaining 403 markers were screened on panels from the other species (*G. tomentosum, G. mustelinum, G. armourianum* or *G. longicalyx*) and 286 (71%) of those were validated to have scoreable genotypes. Sets of markers were also screened which fall under the species-specific data sets, 466 were tested from the *G. tomentosum* set of which 252 were tested on the *G. tomentosum* screening panel which produced 168 (66.7%) successful markers. The other 214 markers were tested on other species and 141 (65.9%) were validated. A total of 138 were tested from the *G. mustelinum* data set, of which 90 were run on the *G. mustelinum* screening panel and 48 were run on other species screening panels. These tests resulted in 61 (67.8%) successful markers from the same-species tests and 27 (56.3%) successful markers from the other-species tests. For the *G. armourianum* data set, 214 markers were tested, of which only 5 were tested on species-specific panels. Overall, 146 out of the 209 (69.9%) markers tested on *G. armourianum* produced successful assays. A small set of 27 markers was tested from the *G. longicalyx* data set, of which 19 (70.4%) generated successful assays. A similar proportion of successful assays was obtained from SNPs generated for *G. longicalyx* using a different *G. hirsutum* assembly version that was abandoned because it yielded poor results when used to define SNPs in other species. Unique SNPs from this set were extracted and included in Additional file 12.

### Validation of *G. barbadense*SNPs – Wide-cross whole-genome radiation hybrid mapping

**Figure 4:**
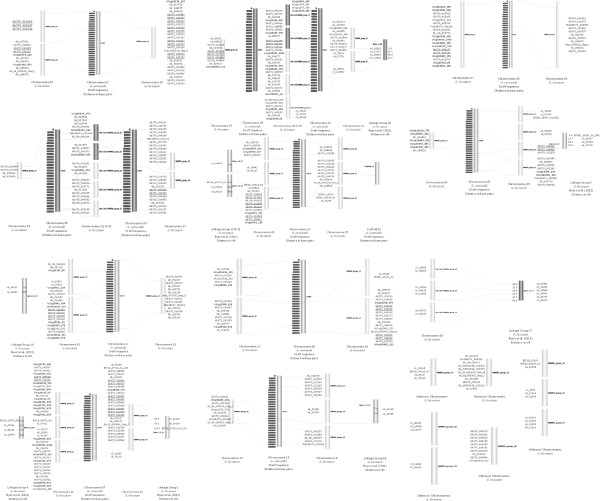
**Wide-cross whole-genome radiation hybrid bin map.** Wide-cross whole-genome radiation hybrid map generated from genotypes of 131 irradiated F1 (*G. hirsutum* line TM-1 × *G. barbadense* cv. 3–79) individuals in Carthagene using LOD score of 3. Bins consist of all markers which fall in a single syntenic group as determined by Carthagene. Bins are aligned to the *G. raimondii* (D_5_) draft genome sequence by BWA mapping of individual SNP markers. Bold markers indicate markers from the Van Deynze et al. [20] data set that were mapped in the Yu et al. [14] paper. Underlined markers indicate markers for which the sequences were overlapped.

**Figure 5:**
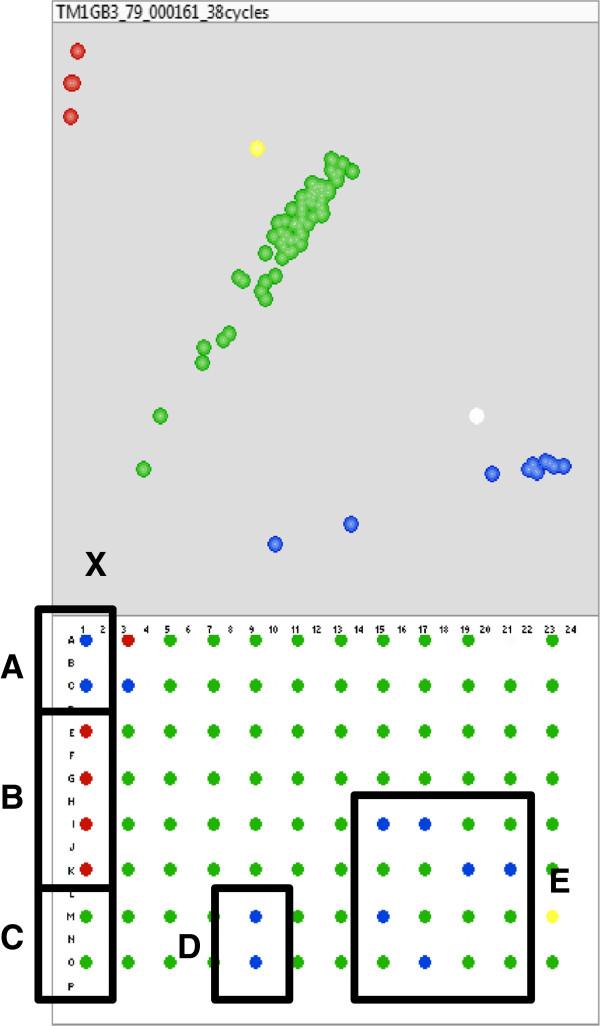
**KASP genotyping of marker UCcg10563_649.** A1 quadrant of a 384-well plate KlusterCaller image of genotyping KASP assay for marker UCcg10563_649 after 38 cycles. **A.)** *G. barbadense L.* (AD)_2_ cv. 3–79, **B.)** *G. hirsutum L.* (AD)_1_ line TM-1, **C.)** F_1_ euploid hybrid *G. barbadense L. (*AD)_2_ cv. 3–79 and *G. hirsutum L.* (AD)_1_ line TM-1, **D.)** F_1_ hypo-aneuploid lines for AD-Chromosome 17, **E.)** Wide-cross whole-genome radiation hybrid samples, **X.)** Non-template (negative control), in B2 quadrant (not shown). Both samples in D and 6 of the samples in E show deletions due to the shift in genotype from F_1_-green to homozygous for the *G. barbadense L. (*AD)_2_ allele – blue. [The two samples shown in white and yellow were considered questionable genotypes as they did not fall directly in a cluster].

A total of 509 markers were WWRH mapped using 131 WWRH individuals (*G. hirsutum* line TM-1 × irradiated pollen of *G. barbadense* cv. 3–79 [16]). Those markers (124) which were found using a previous version of the bioinformatic pipeline (which produced sets with overall lower success rate) and thus are not in the final set (Additional files 2 and 3) have names and sequences listed in Additional file 13. A total of 60 syntenic groups were produced along with 43 singletons (Additional file 14) that were not integrated into a syntenic group. Most of the groups (52) were anchored onto the *G. raimondii* draft genome sequence (Figure 4) by alignment of markers, as well as by chromosome localization using deficiency mapping with F_1_ hypo-aneuploids (Figure 5) and/or the presence of marker(s) that were previously linkage-mapped [14]. The markers in Figure 4 are reported in bins because the order of the markers may not be accurately estimated as the WWRH panel did not provide enough power to precisely order markers.

### Deletion analysis of *G. barbadense*SNPs

The number of WWRH deletions was significantly higher for only 6 (1.2%) of the 509 *G. barbadense* markers used for WWRH mapping, based on Tukey’s boxplot method of outlier detection. These markers were Gb379_011066, Gb379_000467, UCcg10762_238, UCcg10614_274, c4_101926, and c4_44618.

### Functional analysis

A total of 117 contigs containing 118 SNPs (three of the shared SNPs in Figure 1 are loci which have three alleles so these were not included in the analysis) were found to be shared between all wild species relative to the TM-1 *G. hirsutum* reference. When translation was predicted using AUGUSTUS software (http://bioinf.uni-greifswald.de/augustus/) [30] and *Theobroma cacao* as the model species, 52 out of the 116 translations were found to generate different amino acid sequences or non-synonymous substitutions, which corresponds to a Ka/Ks of 0.8125. As a method of comparison to the first set, a random set of 118 SNPs from the overall non-redundant Class I and II data set was chosen. These SNPs represented 118 different contigs. When the same analysis using AUGUSTUS was performed with this random set of contigs, 33 out of the 109 translations were found to generate different amino acid sequences, Ka/Ks of 0.4342.

## Discussion

Interspecific germplasm introgression provides a powerful way of introducing novel beneficial alleles into breeding germplasm of Upland cotton. Its use has been constrained by the long time periods required for introgression and the difficulty of breaking linkage blocks. But when patience is exhibited and recombination has occurred to break linkage blocks to allow for introgression of interspecific segments, highly beneficial products can be obtained. Such is the case with BARBREN which was created to move reniform resistance found in *G. barbadense* into *G. hirsutum* along with superior fiber characteristics [31]. Combining features of *G. barbadense* with *G. hirsutum* has been a long-standing desire, because *G. barbadense* offers many superior fiber trait characteristics but does not produce the high lint yield of traditional *G. hirsutum* cultivars. The large number of *G. barbadense* SNPs identified in this study, 10,521, and particularly the 8,307 Class I and Class II markers will provide markers for a large number of genes in which differences exist between *G. hirsutum* and *G. barbadense*. A modest number, 509 markers, have been validated (from 594 tested) and the majority of these have been anchored to an allotetraploid chromosome by WWRH mapping, therefore relative physical location is known (Figure 4). A larger number of markers and/or larger WWRH panel would have been needed to link all of the singleton markers into the syntenic groups. Markers within each group were close enough for statistical association, but the number and variety of deletions was insufficient to accurately determine physical order. The average number of plants with a deletion for a given SNP was only 8.9 (6.9%), far short of the optimal deletion rate i.e., 50%. Accurate ordering would require a far larger population of similarly irradiated plants or a population with a much higher deletion rate.

Additional investigation into patterns of deletions showed that only 6 (1.2%) of markers across individuals had a statistically significant number of deletions. This was a statistically insignificant number from the overall set. The relative abundance of these marker deletions suggests that the respective chromosomal segments have higher propensities for deletion and/or post-occurrence recovery of induced deletions. Some chromosomal segments may be more likely to be lost after pollen irradiation, and/or there may be significant differences in selection for/against loss of specific genes/alleles in these segments that correspond to the identified markers.

Placement of the syntenic groups relative to the D_5_ reference sequence revealed most to be in non-pericentromeric and non-telomeric regions, i.e., similar to the pattern observed for individual markers (Figure 2). SNPs were distributed unevenly across the chromosomes. A bimodal distribution was observed for each pseudo-chromosome scaffold, with large numbers of SNPs near the subtelomeric regions and small numbers in centromeric and telomeric regions, as would be expected given the metacentric nature of cotton chromosomes and the fact that the markers were derived from expressed sequences [32]. In addition all SNPs were found to integrate into a single syntenic group, unlike SSRs which integrate across groups, which implies the majority of SNPs are subgenome specific and will be useful for breeding as identifying a unique position in the genome.

Mapping experiments were focused on *G. barbadense*. Like Upland cotton, it is a cultivated species and represents approximately five percent of the worldwide cotton production. However, *G. barbadense* being not as highly improved as *G. hirsutum,* it retains some alleles that are deleterious when brought into a *G. hirsutum* background. Some research has been done utilizing chromosome substitution lines, in which a single chromosome in the *G. hirsutum* allotetraploid has been replaced with the same allotetraploid chromosome from a different species, for example *G. barbadense*[33]*.* Many beneficial regions have been identified for yield components as well as fiber quality traits [34, 35]. Recombinant inbred lines from these chromosome substitution lines have also been generated recently that will assist in introgression efforts once markers from a high-density dataset such as was developed here have been located for target areas. It has been shown that cryptic beneficial alleles are typically masked in the overall *G. barbadense* background [36].

Like *G. barbadense*, the other wild allotetraploid species, *G. mustelinum* and *G. tomentosum*, included in this study have also been shown to host cryptic beneficial alleles. These species have been integrated into the chromosome substitution line development effort and will provide additional trait resources for movement into a *G. hirsutum* background. Being of allotetraploid genome constitution, most genomic segments from these species will easily be moved into *G. hirsutum.* Integrating genes from wild diploid species like *G. longicalyx* and *G. armourianum* is much more complicated, because their diploid genomes are vastly different from the *G. hirsutum* genome. However with the longer divergence time and large number of diploid species available, (~45) compared to uncultivated tetraploid species (~3-5), a much larger number of unique beneficial alleles may be found in diploid *Gossypium* species. Inventive methods of creating synthetic polyploids with diploid species have been devised to facilitate transfer of genetic material into *G. hirsutum*, as was the case for *G. longicalyx*, in order to create Upland cottons with strong resistance to reniform nematodes [37]. Two sister lines with strong reniform nematode resistance, LONREN-1 and LONREN-2, were released but subsequently discovered to suffer early growth season “stunting” suggesting a possible linkage drag or pleiotropic effect [38]. In general, practical utilization of introgressed alien germplasm demands precise genetic manipulation to separate linked beneficial and deleterious alien genes, for which numerous markers are essential. Thus, large numbers of markers are needed for each germplasm source, such as the markers developed here. Class I markers will be exceptionally useful as they can be used for determining haplotype information being the only marker identified within a contig.

Studying shared markers between the different species of varied genome composition relative to cultivated *G. hirsutum* can be used to deduce the theoretical ancestral allele at a locus, as well as to suggest functional properties of a locus. The distribution of shared markers is depicted in Figure 1. For markers at which all wild species share a common allele but *G. hirsutum* differs, it can be inferred that the wild species share the ancestral allele and *G. hirsutum* contains an alternative allele. Such alleles are good candidates for being functionally important to domestication, cultivation or agronomic performance, or in linkage disequilibrium with such genes. The non-synonymous to synonymous rate was found to be much higher in the 118 SNPs shared between species (0.8125) than in 118 randomly selection SNPs from the final set (0.4342). This implies that there is a stronger positive selection upon the SNPs where *G. hirsutum* has an allele which is non-ancestral. This further supports the hypothesis that these loci are likely to be important in beneficial traits in *G. hirsutum*.

Some markers within the same species were identified from multiple scaffolds, but were identical in SNP and flanking sequence (Additional files 3, 5, 7, 9, 11). This is likely due to genes from gene families which exhibit very high levels of sequence similarity. Therefore multiple hits are expected for genes that have expanded in the TM-1 or *G. hirsutum* lineage or are duplicated within a genome (paralogs). Another possible explanation is that these hits relate to contigs that contain different isoforms of the same gene. When SNPs derived from multiple isoforms are mapped physically or by linkage, they will locate to a single locus. SNPs from the former case will occupy multiple locations in which different members of the gene family are found. We classified these SNPs separately as they may represent multiple loci throughout the genome and present another level of difficulty for genotyping. The diploid species were found to have many more SNPs exhibiting within-species redundancy, which are marker sequences that are identical but generated from multiple contigs in the assembly, than the tetraploid species. This result was expected because the tetraploid *G. hirsutum* that was utilized as a reference likely received a copy of each gene from ancestors of the A and D subgenomes during polyploidization. Thus relative to each diploid, the reference would have twice as many copies for each gene (assuming no gene loss or duplication has occurred, or co-assembly) and would lead to derivation of the same SNP sequence from each of the homeologous copies found in *G. hirsutum* when analyzing the diploid species*.*

## Conclusions

Markers associated with functional differences between species are essential for generating a feasible system for germplasm introgression via marker-assisted breeding for beneficial agronomic traits. Future large-scale mapping, fine mapping, and genome-wide association analysis efforts to associate the markers developed here as diagnostic markers for traits of interest will allow for marker assisted selection and back crossing to speed up introgression efforts. Advancements in interspecific germplasm introgression are likely to create opportunities for profound improvement of cotton *G. hirsutum* cultivars and will allow for more sustainable provisioning of society in the face of population growth, evolving pathogens and insects, drought and changing environments.

## Methods

### Plant materials

The seed of *G. barbadense* L. (AD)_2_ genetic standard line 3–79, *G. tomentosum* (AD)_3_ plant number 19909036.05 from the Beasley Lab collection, *G. mustelinum* (AD)_4_ plant number 200508123.02 from the Beasley Lab collection, *G. armourianum* (D_2–1_) accession D2-1-6, and *G. longicalyx* (F_1_) plant number 200908137.04 from the Beasley Lab collection were planted at Texas A&M University. Young leaf tissues were sampled from each plant and used to isolate total RNA using the Qiagen RNeasy Mini Kit per manufacturer instructions. RNA isolates were quantified using NanoDrop spectrophotometry (Thermo Scientific, Wilmington, USA) and checked for quality by gel electrophoresis. PolyA RNA was extracted using double purification with oligo dT Dynal beads. Illumina RNA-Seq libraries were prepared using the manufacturer protocol (Illumina Inc, San Diego, USA). Libraries were normalized by denaturation and rehybridization in NaCl and TMAC (tetra-methyl-ammonium-chloride) buffers [39] and then treated with Duplex Specific Nuclease to digest cDNAs from highly abundant transcripts [40]. The treated library was then re-amplified for 12 PCR cycles using Illumina library primers. The libraries were then single-read sequenced using the Illumina Genome Analyzer II for 85 cycles (Table 1). Raw single-read sequence files were uploaded to NCBI under BioProject PRJNA203021 and SRA numbers (SRX457172 – *G. barbadense*, SRX472724 - *G. tomentosum*, SRX - 474879/SRR174699 *G. mustelinum*, SRX474240/SRR1174039 and SRR1174041- *G. armourianum*, and SRX474242/SRR1174179 and SRR1174182 - *G. longicalyx*).

Wide-cross whole-genome radiation (WWRH) individuals [16] were planted and maintained at Texas A&M University. Small leaves were collected from each plant and extracted using the Qiagen DNeasy plant extraction kit per manufacturer instructions.

### SNP development

Reads from each species were trimmed for quality and then aligned to the *G. hirsutum* L. assembly created from genetic standard line, TM-1 (GALV00000000.1 Ashrafi et al. - in preparation), using CLC Genomics Workbench (V5.0). Reads which mapped to multiple locations were randomly assigned to a single location. Putative SNPs between TM-1 and each species were identified one accession at a time. The mapping data were exported as *BAM* files to a Linux server and SAMtools were used to call variants. Subsequent rounds of parameter tweaking resulted in the final pipeline used for SNP development. The resulting pileup files were filtered using the filter pileup Perl script in Galaxy (https://main.g2.bx.psu.edu/) [41] to remove indels and positions with less than coverage of 3. The resulting file was then further filtered using an in-house Perl script which required two genotypes to be homozygous for different bases with minimum coverage of 10. Putative SNPs were then removed from the list if they were located within 50 bases of predicted intron-exon boundary on the TM-1 assembly using SGN (http://solgenomics.net/) intron finder tool. SNPs were further filtered to remove theoretical homeo-SNP positions based on allele-SNP calls generated when Illumina TM-1 reads were mapped back to the TM-1 reference.

9SNPs from each species were classified based on identification of additional SNPs. Class I was defined as SNPs from contigs with no other SNP residing within the contig. Class II was defined as SNPs from contigs that contained one or more additional SNP outside of the 50-bp flanking sequences (none within). Class III was defined as SNPs from contigs that contained one or more additional SNPs within the 50-bp flanking sequences.

### Removal of redundant markers

A FASTA file containing all *in silico*-derived SNPs was used for BLAST analysis (v2.2.27) against a FASTA file containing all *G. hirsutum* markers from the Ashrafi et al. (in preparation) dataset. Markers were removed from the *in silico* set if BLAST analysis showed 100% identity over 100% length of the sequence to the *G. hirsutum* data set. The remaining set was then BLASTed against itself to determine identical markers within the set. Markers with hits in other species groups were removed in a hierarchical method, leaving markers in the highest set, based on the hierarchy *G. barbadense, G. tomentosum, G. mustelinum, G. armourianum,* and *G. longicalyx.* Markers with hits within the same species were separated into a data set containing “overlap markers” (Additional files 3, 5, 7, 9, 11). The result was a final non-redundant data set of Class I and Class II markers for each species (Additional files 1, 4, 6, 8, and 10). The final non-redundant set of Class I and Class II SNPs for each species were submitted to NCBI’s dbSNP (ss974702651-ss974710721 for *G. barbadense,* ss1026506434-ss1026511516 for *G. tomensotum,* ss1026511517-ss1026516811 for *G. mustelinum,* ss1026516812-ss1026536246 for *G. armourianum,* and ss1026536247-ss1026565496 for *G. longicalyx*). Non-redundant Class III SNPs were compiled into Additional file 2 for all species.

### Alignment of markers to D_5_-reference genome

The non-redundant SNPs and overlap markers were aligned to the *G. raimondii* (D_5_) reference genome sequence [27] using Burrows-Wheeler Aligner (BWA) in Galaxy [41] using default settings. Alignment positions were corrected to note the SNP base positions. SNPs were separated into files based on D_5_ genome scaffold alignment. Scaffold files were sorted by genome position. Densities of markers along the D_5_ genome scaffolds were plotted using densityPlot in the R program [42] over scaffold position.

### SNP validation

Subsets of SNPs from the subsequent rounds of bioinformatic filtering were selected for experimental testing using the LGC KASP assays (Beverley, USA). Assay primers were developed using BatchPrimer3 with an optimal primer Tm of 57°C (minimum 55°C, maximum 60°C, maximum difference between primers of 5°C), optimal product size of 50 base pairs (minimum 50 base pairs, maximum 100 base pairs) and the default settings were used for the remaining parameters. Primers were mixed at the dilutions specified by LGC then used to perform KASP assays on small screening panels containing duplicates, e.g., the panel used to screen *G. barbadense* markers contained 2 *G. hirsutum L.* TM-1, 2 *G. barbadense* 3*–*79, 2 euploid F1 (*G. hirsutum x G. barbadense*), 4 RILs from a *G. hirsutum × G. barbadense* mapping population, and 2 non-template (negative) controls (Figure 3). Plates were initially run for the recommended 38 cycles on the LGC SNP platform, centrifuged then read on the Pherastar plate reader. The Pherastar files were imported into KlusterCaller software for genotyping (Figure 5). If the plates were determined to be insufficiently clustered, additional sets of 3 cycles were added and the plates were re-read and re-imported, until scoreable clusters were formed or the marker was deemed to be unacceptable.

### Wide-cross whole-genome radiation hybrid mapping

Those *G. barbadense* markers which clustered well from all rounds of development used for parameter tweaking until the final pipeline was reached were then run on a “full-panel” containing duplicates of the parental and F_1_ controls (*G. hirsutum* line TM-1, *G. barbadense* cultivar 3–79, euploid F1), *G. hirsutum* cultivar DP-90, *G. barbadense* cultivar Phytogen800, 131 wide-cross whole-genome radiation hybrid individuals, 47 F_1_ hypo-aneuploid lines, and 4 non-template negative controls. Genotypes were manually curated. All questionable genotypes were listed as unscored. A shift of genotype from the F1 heterozygote cluster to the homozygous 3–79 cluster was interpreted as a deletion in the respective interspecific WWRH or hypo-aneuploid F_1_ cytogenetic stock (Figure 1). Genotype files were manipulated to note presence (1) or absence (0) of a deletion in the WWRH plants.

An additional set of markers from two previous studies, Van Deynze et al. [20] and Byers et al. [19], were also genotyped using KASP assays. Genotypes for these markers were also manipulated to note presence or absence of a deletion in the WWRH plants.

SNP markers which showed no deletions among the 131 WWRH samples were removed from the WWRH mapping analysis. Genotype files in binary format were analyzed using Carthagene, with a LOD score of 3.0 and mapping distance within 100 cR. From the resulting syntenic groups, the singleton groups were removed and classified as non-linked markers (Additional file 14). Those syntenic groups with two or more markers were subjected to finishing methods using annealing, flips and polishing to determine the final group order. The resulting syntenic groups were cross-referenced with the D_5_ alignments of individual markers, as well as the chromosome locations determined by deletion analysis with the F_1_ hypo-aneuploids. Syntenic groups and their relationships based on alignment to the D_5_ scaffolds were plotted (Figure 4) using Strudel software [43].

### Deletions analysis

The numerical distribution of deletions in the radiation hybrids per marker was analyzed by a box plot method to determine the number of deletions that would be statistically significant. The first and third quartile were determined along with the mean, then the inner quartile range (IQR) was utilized to calculate the threshold for outliers greater than 1.5 times the IQR. Markers which had numbers of deletions beyond the threshold were determined to have a significantly different number of deletions than expected.

### Functional analysis

Contigs for which the SNPs represented identical differences from *G. hirsutum* TM-1 for all five species from the reference were parsed into a FASTA file containing 117 contigs for 118 SNP. The reference FASTA file was modified to contain the alternate SNP allele(s) using an in house Perl script. Both the reference and alternate FASTA files were analyzed using AUGUSTUS [30] to predict translation start and end sites using *Theobroma cacao* as the model species. Non-synonymous and synonymous changes between the reference and alternate files were calculated. Predicted amino acid coding sequences were then investigated using TBLASTX against NCBI’s non-redundant database. BLAST results were parsed to contain the top hit with covcutoff of 50 and *e* value cut off of *1*^*e-8*^. The differences between predicted protein products were investigated. The same analysis was performed using a randomly selected set of 118 SNP from the final overall Class I and Class II data set.

## Availability of supporting data

The data sets supporting the results of this article are included within the article and its additional files.

## Electronic supplementary material

Additional file 1: ***G. barbadense*** **cv. 3–79 SNPs relative to** ***G. hirsutum*** **line TM-1.** SNPs and 50-bp flanking sequences for *in silico* derived SNPs in *G. barbadense* cv. 3–79 relative to *G. hirsutum* line TM-1. SNPs have been classified into two categories, Class I are SNPs from contigs with no other SNP residing within the contig. Class II are SNPs from contigs that contain one or more additional SNP outside of the 50-bp flanking sequences (none within). Columns 4 and 5: Alignment position and scaffold information to *G. raimondii* (D_5_) draft genome sequence using BWA. Column 6 indicates overlap information for *G. barbadense* markers with *G. tomentosum, G. mustelinum, G. armourianum, and G. longicalyx*, where the Arabic numeral indicates number of shared species and subsequent abbreviations indicate with which of the above species the marker is shared (Gb, Gt, Gm, Ga and Gl, respectively). (XLSX 1023 KB)

Additional file 2: **Five species Class III SNPs. Class III SNPs for** ***G. barbadense*** **cv. 3–79,** ***G. tomentosum, G. mustelinum, G. armourianum,*** **and** ***G. longicalyx.*** Class III are SNPs from contigs that contain one or more additional SNPs within the 50-bp flanking sequences. Class III SNP sequences are provided for reference, but have been shown to have issues during experimental screening using KASP assays. (XLSX 1 MB)

Additional file 3: ***G. barbadense*** **cv. 3–79 SNPs relative to** ***G. hirsutum*** **line TM-1 within-species overlapping markers.** SNPs and 50-bp flanking sequences for *in silico*-derived SNPs of *G. barbadense* (cultivar 3–79) relative to *G. hirsutum* (genetic standard line TM-1) that have been found to be identical for SNP and flanking sequence within the *G. barbadense* data set. SNPs have been classified into two categories, Class I are SNPs from contigs with no other SNP residing within the contig. Class II are SNPs from contigs that contain one or more additional SNP outside of the 50-bp flanking sequences (none within). Alignment position and scaffold information to *G. raimondii* (D_5_) draft genome sequence using BWA. Column 6 indicates overlap information for *G. barbadense* markers with *G. tomentosum, G. mustelinum, G. armourianum, and G. longicalyx*, where the Arabic numeral indicates number of shared species and subsequent abbreviations indicate with which of the above species the marker is shared (Gb, Gt, Gm, Ga and Gl, respectively). (XLSX 101 KB)

Additional file 4: ***G. tomentosum*** **SNPs relative to** ***G. hirsutum*** **line TM-1.** SNPs and 50-bp flanking sequences for *in silico*-derived SNPs of *G. tomentosum* relative to *G. hirsutum* line TM-1. SNPs have been classified into two categories, Class I are SNPs from contigs with no other SNP residing within the contig. Class II are SNPs from contigs that contain one or more additional SNP outside of the 50-bp flanking sequences (none within). Alignment position and scaffold information to *G. raimondii* (D_5_) draft genome sequence using BWA. Column 6 indicates overlap information for *G. tomentosum* markers with *G. mustelinum, G. armourianum, and G. longicalyx*, where the Arabic numeral indicates number of shared species and subsequent abbreviations indicate with which of the above species the marker is shared (Gt, Gm, Ga and Gl, respectively). (XLSX 487 KB)

Additional file 5: ***G. tomentosum*** **SNPs relative to** ***G. hirsutum*** **line TM-1 within species overlapping markers.** SNPs and 50-bp flanking sequences for *in silico*-derived SNPs of *G. tomentosum* relative to *G. hirsutum* line TM-1 that have been found to be identical for SNP and flanking sequence within the *G. tomentosum* data set. SNPs have been classified into two categories, Class I are SNPs from contigs with no other SNP residing within the contig. Class II are SNPs from contigs that contain one or more additional SNP outside of the 50-bp flanking sequences (none within). Alignment position and scaffold information to *G. raimondii* (D_5_) draft genome sequence using BWA. Column 6 indicates overlap information for *G. tomentosum* markers with *G. mustelinum, G. armourianum, and G. longicalyx*, where the Arabic numeral indicates number of shared species and subsequent abbreviations indicate with which of the above species the marker is shared (Gt, Gm, Ga and Gl, respectively). (XLSX 60 KB)

Additional file 6: ***Gossypium mustelinum*** **SNPs relative to** ***G. hirsutum*** **line TM-1.** SNPs and 50-bp flanking sequences for *in silico*-derived SNPs of *G. mustelinum* relative to *G. hirsutum* line TM-1. SNPs have been classified into two categories, Class I are SNPs from contigs with no other SNP residing within the contig. Class II are SNPs from contigs that contain one or more additional SNP outside of the 50-bp flanking sequences (none within). Alignment position and scaffold information to *G. raimondii* (D_5_) draft genome sequence using BWA. Column 6 indicates overlap information for *G. mustelinum* markers with *G. armourianum, and G. longicalyx*, where the Arabic numeral indicates number of shared species and subsequent abbreviations indicate with which of the above species the marker is shared (Gm, Ga and Gl, respectively). (XLSX 487 KB)

Additional file 7: ***G. mustelinum*** **SNPs relative to** ***G. hirsutum*** **line TM-1 within species overlapping markers.** SNPs and 50-bp flanking sequences for *in silico*-derived SNPs of *G. mustelinum* relative to *G. hirsutum* line TM-1 that have been found to be identical for SNP and flanking sequence within the *G. mustelinum* data set. SNPs have been classified into two categories, Class I are SNPs from contigs with no other SNP residing within the contig. Class II are SNPs from contigs that contain one or more additional SNP outside of the 50-bp flanking sequences (none within). Alignment position and scaffold information to *G. raimondii* (D_5_) draft genome sequence using BWA. Column 6 indicates overlap information for *G. mustelinum* markers with *G. armourianum, and G. longicalyx*, where the Arabic numeral indicates number of shared species and subsequent abbreviations indicate with which of the above species the marker is shared (Gm, Ga and Gl, respectively). (XLSX 58 KB)

Additional file 8: ***G. armourianum*** **SNPs relative to** ***G. hirsutum*** **line TM-1.** SNPs and 50-bp flanking sequences for *in silico*-derived SNPs of *G. armourianum* relative to *G. hirsutum* line TM-1. SNPs have been classified into two categories, Class I are SNPs from contigs with no other SNP residing within the contig. Class II are SNPs from contigs that contain one or more additional SNP outside of the 50-bp flanking sequences (none within). Alignment position and scaffold information to *G. raimondii* (D_5_) draft genome sequence using BWA. Column 6 indicates overlap between the two species *G. armourianum* markers with *G. longicalyx*. (XLSX 2 MB)

Additional file 9: ***G. armourianum*** **SNPs relative to** ***G. hirsutum*** **line TM-1 within species overlapping markers.** SNPs and 50-bp flanking sequences for *in silico*-derived SNPs of *G. armourianum* relative to *G. hirsutum* line TM-1 that have been found to be identical for SNP and flanking sequence within the *G. armourianum* data set. SNPs have been classified into two categories, Class I are SNPs from contigs with no other SNP residing within the contig. Class II are SNPs from contigs that contain one or more additional SNP outside of the 50-bp flanking sequences (none within). Alignment position and scaffold information to *G. raimondii* (D_5_) draft genome sequence using BWA. Column 6 indicates overlap between the two species *G. armourianum* markers with *G. longicalyx*. (XLSX 220 KB)

Additional file 10: ***G. longicalyx*** **SNPs relative to** ***G. hirsutum*** **line TM-1.** SNPs and 50-bp flanking sequences for *in silico*-derived SNPs of *G. longicalyx* relative to *G. hirsutum* line TM-1. SNPs have been classified into two categories, Class I are SNPs from contigs with no other SNP residing within the contig. Class II are SNPs from contigs that contain one or more additional SNP outside of the 50-bp flanking sequences (none within). Alignment position and scaffold information to *G. raimondii* (D_5_) draft genome sequence using BWA. (XLSX 2 MB)

Additional file 11: ***G. longicalyx*** **SNPs relative to** ***G. hirsutum*** **line TM-1 within species overlapping markers.** SNPs and 50-bp flanking sequences for *in silico*-derived SNPs of *G. armourianum* relative to *G. hirsutum* line TM-1 that have been found to be identical for SNP and flanking sequence within the *G. armourianum* data set. SNPs have been classified into two categories, Class I are SNPs from contigs with no other SNP residing within the contig. Class II are SNPs from contigs that contain one or more additional SNP outside of the 50-bp flanking sequences (none within). Alignment position and scaffold information to *G. raimondii* (D_5_) draft genome sequence using BWA. (XLSX 295 KB)

Additional file 12: **Additional** ***G. longicalyx*** **SNPs (Class I, Class II & Class III).** “Specific” *G. longicalyx* SNP markers, which were determined using an alternative version of the TM-1 assembly. Markers are classified into three categories, Class I are SNPs from contigs with no other SNP residing within the contig. Class II are SNPs from contigs that contain one or more additional SNP outside of the 50-bp flanking sequences (none within). Class III are SNPs from contigs that contain one or more additional SNPs within the 50-bp flanking sequences. (XLSX 5 MB)

Additional file 13: ***G. barbadense*** **SNPs relative to** ***G. hirsutum*** **line TM-1 markers from different pipelines included in wide-cross whole-genome radiation hybrid mapping.** SNPs and 50-bp flanking sequence for *in silico* derived SNPs in *G. barbadense* relative to *G. hirsutum* (genetic standard line TM-1). SNP and flanking sequence are listed for markers which did not overlap the set of SNPs produced using the final bioinformatic analyses to produce markers in Additional files 1 and 3. (XLSX 21 KB)

Additional file 14: **Singleton markers from** ***G. barbadense*** **wide-cross whole-genome radiation hybrid mapping.** List of markers which did not fall into any syntenic group from Carthagene analysis with *G. barbadense* markers used for whole-genome radiation hybrid mapping. (XLSX 9 KB)

## Abbreviations

SNP: Single nucleotide polymorphism
RFLP: Restriction fragment length polymorphism
AFLP: Amplified fragment length polymorphism
SSR: Simple sequence length repeat
WWRH: Wide-cross whole-genome radiation hybrid
QTL: Quantitative trait loci
MAS: Marker assisted selection.
